# Diversity, distribution and biology of Romanian flat-footed flies (Diptera, Opetiidae and Platypezidae) with taxonomic notes on *Callomyia saibhira* Chandler

**DOI:** 10.3897/zookeys.459.8376

**Published:** 2014-12-02

**Authors:** Michal Tkoč, Jindřich Roháček

**Affiliations:** 1 Department of Entomology, National Museum, Cirkusová 1740, CZ-193 00 Praha 9 – Horní Počernice, Czech Republic; 2 Department of Zoology, Faculty of Science, Charles University in Prague, Viničná 7, CZ-128 43 Praha 2, Czech Republic; 3 Silesian Museum, Nádražní okruh 31, CZ-746 01 Opava, Czech Republic

**Keywords:** Diptera, Opetiidae, Platypezidae, *Callomyia saibhira* redescription, Palaearctic Region, Romania, distribution, biodiversity, new records, biology

## Abstract

Altogether 18 species of the families Opetiidae and Platypezidae are reported from Romania, based on newly studied material and previously published records. The following three species are recorded from Romania for the first time: *Agathomyia vernalis* Shatalkin, 1981, *Callomyia saibhira* Chandler, 1976, and *Lindneromyia hungarica* Chandler, 2001. The presented differential diagnosis and a detailed redescription of body and genitalia of the male of *Callomyia saibhira* are based on one specimen which is the first male found in Europe. Information about distribution and biology of all 18 Romanian species is provided as well as photographs of selected important species. Finally, a new checklist of all Romanian species is given.

## Introduction

The Opetiidae and Platypezidae are basal cyclorrhaphous families of Diptera, belonging to the superfamily Platypezoidea. The European species are small brachycerous flies, ranging from 1.4 to 6.0 mm in wing length. Their coloration is often black (males) or composed of black, orange and grey (females), and some species have silvery grey reflective patterns. The males have larger heads with holoptic eyes, while the female eyes are dichoptic. The larvae may be flat or cylindrical. All known larvae are mycophagous and feed by burrowing in the tissue of fungus fruiting bodies, at the surface of the gills of gill fungi, or on fungal mycelia under bark of dead trees; one species, *Agathomyia wankowiczii* (Schnabl, 1884), is gall-forming on sporocarps of a polypore. Adults of European species may be observed running rapidly on broad leaves in forested habitats; females may be observed ovipositing on host fungi.

These two families of flat-footed flies include 44 species in 13 genera in Europe (Chandler 2001, 2004). Current literature (Chandler 2001, 2004) lists only six species from Romania. However, during the preparation of this paper we have noticed that some localities of Platypezidae given by Thalhammer (1899) and Szilády (1941) are in fact in the present territory of Romania. Unfortunately, the material of Thalhammer (1899) and Szilády (1941) was destroyed by fire in 1956 (Papp 2001), but we consider their determinations to be correct and thus their records are included in the present paper. The monograph of the European species by Chandler (2001) summarizes all known (up to year 2000) data on adult and larval morphology, biology, distribution, systematics, including keys to the species. The nomenclature and classification used here therefore follow Chandler (2001).

## Material and methods

Specimens were examined with an Olympus SZX10 binocular microscope. Photographs were taken by Canon 600D and/or 60D with MPE-65 macro lens and in some cases combined from multiple layers using Helicon Focus Pro 5.2. Drawings and photographs were edited in CorelDRAW 12 and Corel PHOTO-PAINT 12 graphic software. Morphological terminology follows Cumming and Wood (2009) and Chandler (2001), terminology of male genitalia follows Chandler (2001), Chandler and Shatalkin (1998), and is supplemented in parentheses by terminology adopted from Cumming and Wood (2009). The material examined is now deposited in the Silesian Museum, Opava, Czech Republic (SMOC, all specimens collected by J. Roháček) and the National Museum, Praha, Czech Republic (NMPC, remaining specimens).

Distributional data follows Chandler (2001, 2004) and are supplemented by data of Thalhammer (1899), Szilády (1941), Carles-Tolrá and Báez (2002), Ševčík (2004), Pakalniškis et al. (2006), Schacht (2006), Ståhls and Kahanpää (2006), Tkoč and Vaňhara (2006, 2008), Roháček and Ševčík (2007, 2011), Vaňhara (2009), Andrade and Almeida (2010), Ebejer and Andrade (2010), Tkoč (2011), Tkoč et al. (2012), Claussen (2013) and Ståhls (2014).

The following abbreviations are used in the text: I–XII – January to December, BMNH – The Natural History Museum, London, United Kingdom, JR – Jindřich Roháček, ER – European Russia, FE – Far East of Russia, MT – Michal Tkoč, NMPC – National Museum, Praha, Czech Republic, SMNS – Staatliches Museum für Naturkunde, Stuttgart, Germany, SMOC – Silesian Museum, Opava, Czech Republic. The species with asterisk (*) in front of their names represent new records for Romania. The translations of original localities from Romanian are in square brackets [ ], together with names of the respective county and historical region.

## Results

### Family Opetiidae

#### Opetia nigra

Taxon classificationAnimaliaDipteraOpetiidae

Meigen, 1830

Figure 1

##### Published records.

Orlát [Orlat, Sibiu, Transilvania] (Thalhammer 1899); Mehádia [Mehadia, Caraș-Severin, Banat] (Szilády 1941).

##### Material examined.

1 ♂, 1. vi. 2008, Banat, Sfânta Elena, 4km NE, Kulhavá skála, Vranovec cave (Figure 17), 300 m a.s.l., 44°42'12"N, 21°43'52"E, sweeping vegetation along brook, JR leg.

##### Distribution.

Palaearctic species. Recorded in Austria, Belgium, the Czech Republic, Denmark, Finland, France, Germany, Great Britain, Hungary, Ireland, Italy, Luxembourg, the Netherlands, Poland, Portugal, Romania, Slovakia, Spain, Sweden, Switzerland and Russia (ER).

##### Biology.

The adults run on broad leaves in wooded biotopes, where they sometimes form swarms. Its larvae are unknown. The adults were reared from very rotten beech wood and leaf litter (Speight et al. 1990, Chandler 2001) but the exact development substrate of the larvae remains unknown and thus these records need confirmation. The males can sometimes be caught by light trap (Chandler 2001), while the females can be collected by pitfall traps (Vaňhara 1986). The adults (mostly males) can be collected by sweeping undergrowth of various forests, sweeping on *Atropa belladonna* leaves proved to be particularly productive (pers. obs.). The species is bivoltine, adult flight period in central Europe is V–VI and VIII–X.

**Figure 1.**
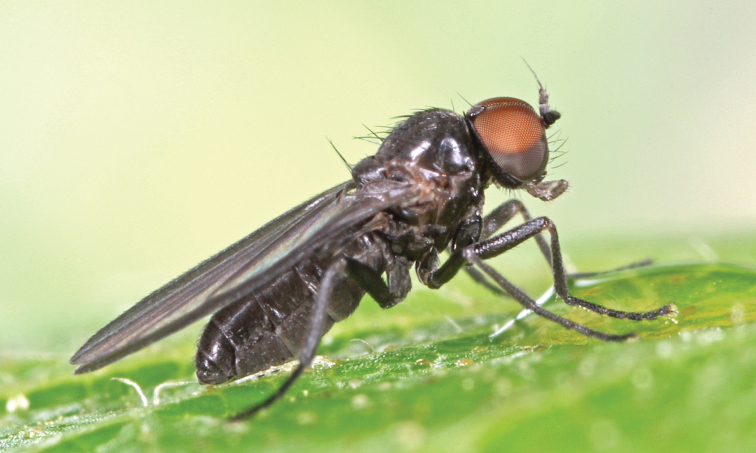
*Opetia nigra* Meigen, 1830, male habitus. Photo by J. Roháček.

### Family Platypezidae

#### Subfamily Callomyiinae

##### Agathomyia antennata

Taxon classificationAnimaliaDipteraPlatypezidae

(Zetterstedt, 1819)

Figure 2

###### Published records.

Mehádia [Mehadia, Caraș-Severin, Banat]; Szászka [Szászka, Caraș-Severin, Banat] (Szilády 1941).

###### Material examined.

1 ♀, 31. v. 2008, Banat, Sfânta Elena, 1 km E, Alibeg brook valley (Figure 18), 230 m a.s.l., 44°40'37"N, 21°43'32"E, sweeping undergrowth of deciduous forest, JR leg.; 3 ♀♀, 1. vi. 2008, Banat, Sfânta Elena, 4 km NE, Kulhavá skála, Vranovec cave (Figure 17), 300 m a.s.l., 44°42'12"N, 21°43'52"E, sweeping vegetation along brook, JR leg.; 1 ♀, 4. vi. 2008, Banat, Berzasca, 2 km NE, Berzasca river valley, 85 m a.s.l., 44°39'09"N, 21°57'47"E, sweeping riverside vegetation, JR leg.; 1 ♀, 19. v. 2011, Alba, Alba Iulia, 1 km E, 380 m a.s.l., 46°04'18"N, 23°32'02"E, sweeping on *Quercus* sp., MT leg.

###### Distribution.

Palaearctic species reaching to Oriental region (Taiwan). Recorded in Austria, Belgium, Croatia, the Czech Republic, Denmark, Finland, France, Germany, Great Britain, Hungary, Italy, Lithuania, the Netherlands, Norway, Poland, Portugal, Romania, Slovakia, Spain, Sweden, Switzerland and Russia (ER, FE).

###### Biology.

Most common species of the genus in the Palaearctic Region. The adults of both sexes are often found running on *Petasites* sp. leaves (pers. obs.). Larvae develop in *Bjerkandera adusta* (Ševčík 2010). Adult flight period is in IV–IX.

**Figure 2.**
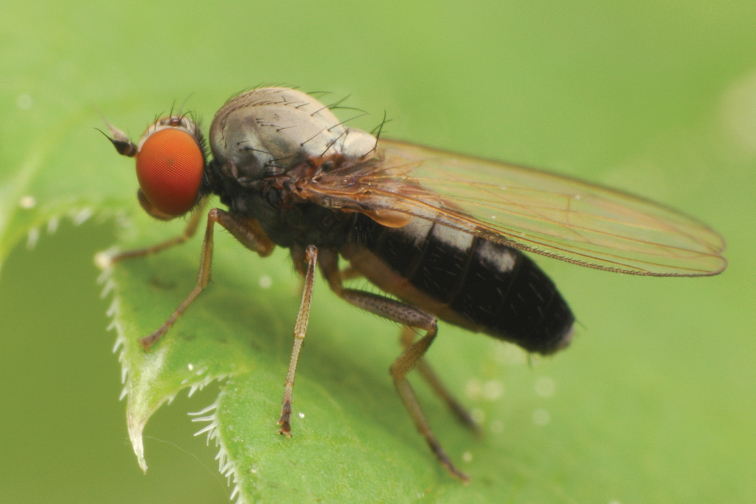
*Agathomyia antennata* (Zetterstedt, 1819), female habitus. Photo by D. Gavryushin.

##### Agathomyia collini

Taxon classificationAnimaliaDipteraPlatypezidae

Verrall, 1901

###### Published records.

Mehadia, Karaš-Severin [Caraș-Severin, Banat], 3.vii.1912, Oldenberg coll. (SMNS) (Czerny 1930, Chandler 2001).

###### Distribution.

Palaearctic species. Recorded in the Czech Republic, France, Great Britain, Hungary, Romania, Slovakia, Spain, Russia (ER, FE) and Georgia (North Ossetia) in the Caucasus (Shatalkin 1992).

###### Biology.

Larvae are unknown; Chandler (2001) mentioned possible association with *Phellinus pomaceus* growing on apple or plum trees based on information provided by Morley (1918). However, this host association needs confirmation. The adults can be swept on forest undergrowth formed mainly by *Lunaria rediviva* (Roháček and Ševčík 2011; pers. obs.). Adults occur in IV–IX.

##### Agathomyia falleni

Taxon classificationAnimaliaDipteraPlatypezidae

(Zetterstedt, 1838)

Figure 3

###### Published records.

Szászka [Szászka, Banat, Caraș-Severin] (Szilády 1941).

###### Distribution.

Palaearctic species. Recorded in Austria, Croatia, the Czech Republic, Denmark, Finland, France, Germany, Great Britain, Hungary, Lithuania, the Netherlands, Romania, Poland, Slovakia, Sweden, Switzerland and Russia (ER).

###### Biology.

**Figure 3.**
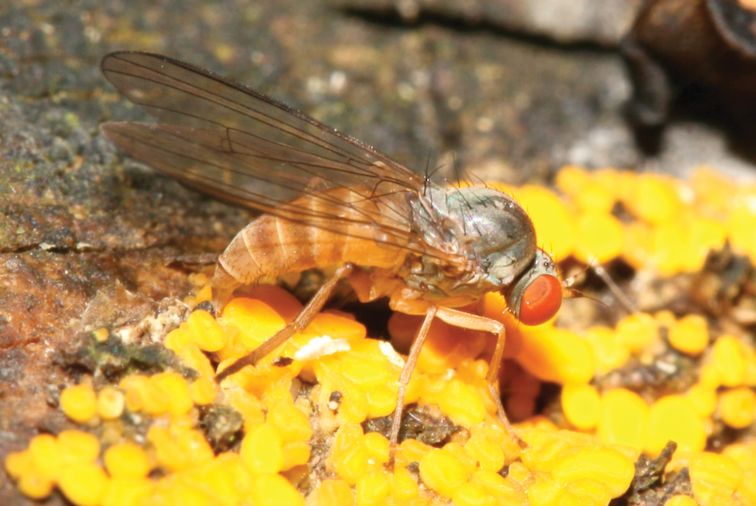
*Agathomyia falleni* (Zetterstedt, 1838), female ovipositing on undetermined yellow slime mold (Mycetozoa) on stump overgrown by *Bjerkandera adusta*. Photo by M. Tkoč.

Host fungus is *Bjerkandera adusta* (see Chandler 2001). The females can be observed during oviposition on sporocarps of the host fungi on tree stumps (Figure 3). It is a species with autumnal activity; adult flight period is in IX–XI.

##### Agathomyia setipes

Taxon classificationAnimaliaDipteraPlatypezidae

Oldenberg, 1916

Figure 4

###### Published records.

1 ♂, Czerna Ufers [=Cerna river banks], Herkulesbad [Băile Herculane, Caraș-Severin, Banat], 13.vii.1912 (Oldenberg 1916, Czerny 1930).

###### Distribution.

Palaearctic species. Recorded in Croatia, the Czech Republic, Slovakia, Hungary, Romania and Russia (FE).

###### Biology.

Unknown. Adults are usually swept from vegetation along brooks in forests (Roháček and Ševčík 2009). Very rare species known from only a few specimens from the whole of Europe; adult flight period is in VII–X.

**Figure 4.**
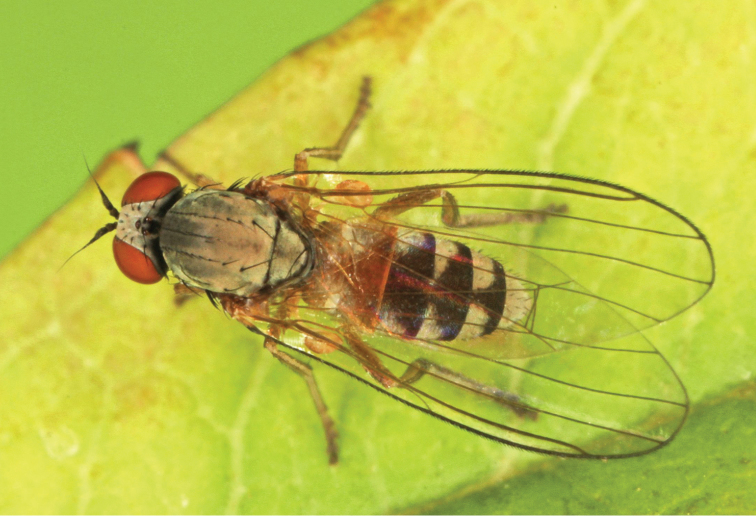
*Agathomyia setipes* Oldenberg, 1916, female showing abdominal pattern. Photo by J. Roháček.

##### * Agathomyia vernalis

Taxon classificationAnimaliaDipteraPlatypezidae

Shatalkin, 1981

Figure 5

###### Material examined.

1 ♀, 19. v. 2011, Alba, Alba Iulia, 1 km E, 380 m a.s.l., 46°04'18"N, 23°32'02"E, sweeping on *Fagus sylvatica*, MT leg.

###### Distribution.

Palaearctic species. Recorded in the Czech Republic, Finland, Germany, Slovakia, Switzerland and Russia (ER). **New record for Romania.**

###### Biology.

Very rare species with early flight period. The individuals are collected only in IV and V and were almost exclusively females. Larval biology and host fungus are unknown. Tkoč and Barták (2013) recorded this species in numbers from a pyramidal (emergence) trap baited with dead wood.

**Figure 5.**
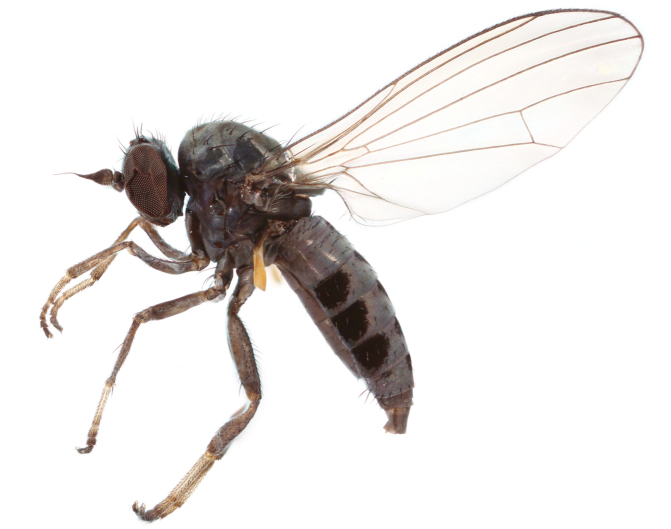
*Agathomyia vernalis* Shatalkin, 1981, female habitus in lateral view. Photo by M. Tkoč.

##### Agathomyia viduella

Taxon classificationAnimaliaDipteraPlatypezidae

(Zetterstedt, 1838)

Figure 6

###### Published records.

Mehádia [Mehadia, Caraș-Severin, Banat] (Szilády 1941).

###### Material examined.

1 ♀, 1. vi. 2008, Banat, Sfânta Elena, 4 km NE, Kulhavá skála, Vranovec cave (Figure 17), 300 m a.s.l., 44°42'12"N, 21°43'52"E, sweeping vegetation along brook, JR leg.

###### Distribution.

Palaearctic species. Recorded in the Czech Republic, Denmark, Finland, France, Germany, Great Britain, Hungary, Ireland, Lithuania, Montenegro, the Netherlands, Norway, Poland, Romania, Slovakia, Sweden, Switzerland and Russia (ER, FE).

###### Biology.

Uncommon species with adults occurring in undergrowth and along brooks in humid deciduous and mixed forests (Roháček and Ševčík 2009) and are often observed running on *Petasites* sp. leaves together with *Agathomyia antennata* (pers. obs.). The main flight period of adults ranges from V to VII. Host fungus is unknown.

**Figure 6.**
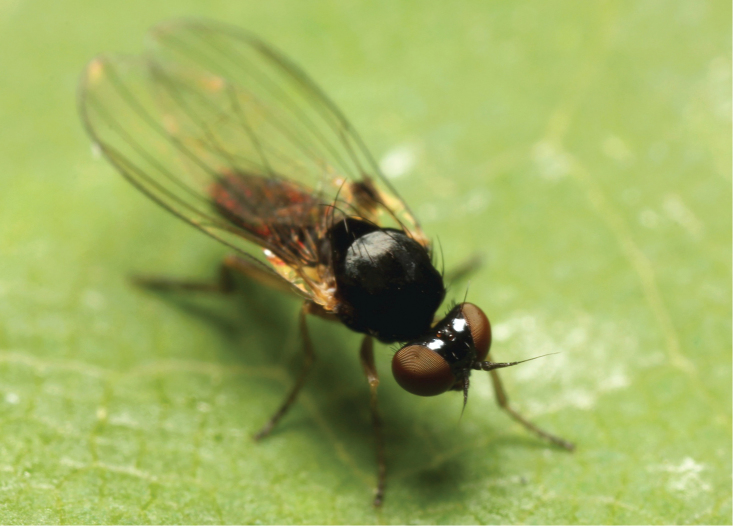
*Agathomyia viduella* (Zetterstedt, 1838), female habitus. Note the glossy frons as the main diagnostic character of females of this species. Photo by M. Tkoč.

##### Callomyia amoena

Taxon classificationAnimaliaDipteraPlatypezidae

Meigen, 1824

Figure 7

###### Published records.

Mehádia [Mehadia, Caraș-Severin, Banat]; Orsova [Orșova, Mehedinți, Banat] (Thalhammer 1899); 1 ♂, 1 ♀, Retezatului Mts, near Hobita, Calana [Hobița, Hunedoara], 29. vi. 1969, in mature pine forest, B.H. Cogan and R.I. Vane-Wright leg. (BMNH) (Chandler 2001, in litt.).

###### Material examined.

1 ♀, 1 vi. 2008, Banat, Sfânta Elena, 4 km NE, Kulhavá skála, Vranovec cave (Figure 17), 300 m a.s.l., 44°42'12"N, 21°43'52"E, sweeping vegetation along brook, JR leg.; 1 ♂ 1 ♀, 2. vi. 2008, Banat, Radimna near Pojejena, 140 m a.s.l., 44°49'17"N, 21°33'31"E, sweeping undergrowth of alder forest, JR leg.; 1 ♂, 19. v. 2011, Alba, Alba Iulia, 1 km E, 380 m a.s.l., 46°04'18"N, 23°32'02"E, sweeping on *Acer campestre*, MT leg.

###### Distribution.

Palaearctic species. Recorded in Austria, Belgium, the Czech Republic, Denmark, Finland, France, Germany, Great Britain, Hungary, Ireland, Italy, Lithuania, the Netherlands, Norway, Poland, Slovakia, Spain, Sweden, Switzerland, Romania and Russia (ER).

###### Biology.

Common species, the larvae live on mycelia under bark of fallen trunks of various trees. A record from mycelia on bark on the underside of aspen trunks lying on the ground was mentioned by Krivosheina (2008). Flight period of adults ranges from V to X.

**Figure 7.**
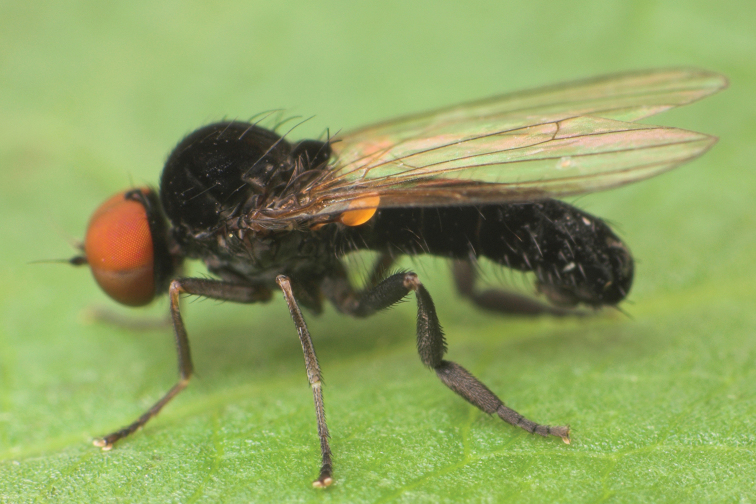
*Callomyia amoena* Meigen, 1824, male habitus. Photo by D. Gavryushin.

##### Callomyia elegans

Taxon classificationAnimaliaDipteraPlatypezidae

Meigen, 1804

Figure 8

###### Published records.

Mehádia [Mehadia, Caraș-Severin, Banat] (Thalhammer 1899).

###### Distribution.

Palaearctic species. Recorded in Andorra, Austria, the Czech Republic, Finland, France, Germany, Great Britain, Hungary, Ireland, Italy, Lithuania, the Netherlands, Norway, Poland, Slovakia, Sweden, Switzerland, Romania and Russia (ER).

###### Biology.

Unknown, the larvae probably live on mycelia under bark of fallen trunks of various trees (similarly to other species of *Callomyia*, see Krivosheina 2008). Rare species with no recent records from Central and South Europe, population densities of this species are very low or undetectable. Adult flight period is in IV–VIII.

**Figure 8.**
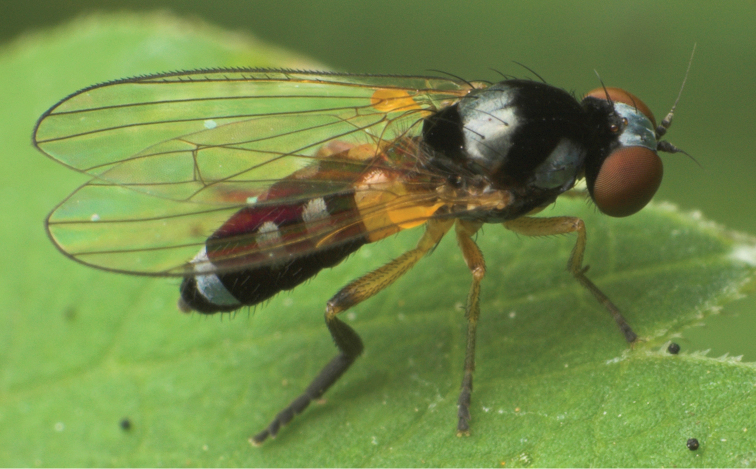
*Callomyia elegans* Meigen, 1804, female habitus. Photo by D. Gavryushin.

##### * Callomyia saibhira

Taxon classificationAnimaliaDipteraPlatypezidae

Chandler, 1976

Figs 9 –10

###### Material examined.

1 ♂, 1. vi. 2008, Banat, Sfânta Elena, 4 km NE, Kulhavá skála, Vranovec cave (Figure 17), 300 m a.s.l., 44°42'12"N, 21°43'52"E, sweeping vegetation along brook, JR leg.

**Differential diagnosis.** Male of *Callomyia saibhira* differs from *Callomyia amoena* and *Callomyia elegans* by having darker halteres that are not orange (brown with knob black). *Callomyia speciosa* have longer arista and shorter first flagellomere and upper part of pleura is not so silvery grey dusted as in *Callomyia saibhira*. From its most similar species, *Callomyia dives* Zetterstedt, 1838, it differs by a clear wing membrane, brown palpus and different genitalia, basal lobe of gonopod is shorter (Figure 10). Females have unique abdominal coloration: tergites 1–4 (T1–4) are orange yellow with narrow brown hind margins on T2–4, only T5 is black, T6 and terminal segments are silvery grey dusted.

###### Redescription.

***Male.*** Body length 4.1 mm. Wing length 3.8 mm.

*Head* black with silvery grey dusting. Antenna dark brown, scapus with dorsal seta reaching to tip of pedicel, pedicel with one strong dorsal seta reaching to the middle of first flagellomere, both sides of pedicel with 3 short setae, 1 short seta on ventral position. First flagellomere conical, twice as long as pedicel. Second and third flagellomere long. Arista half of antennal length. Two pairs of small frontal setae. Ocellar tubercle dull brown, with one pair of ocellar setae and one pair of small postocellar setae. Postocular setae long, their apices visible in anterior view. Face and parafacial bare, silvery grey dusted. Gena, occiput and postgena with long black setae. Occiput black, silvery grey dusted. Palpi brown with short black setae, proboscis brown with pale pubescence.

*Thorax* velvet black with silvery grey dusted areas. Two very inconspicuous median dorsal grey stripes between dorsocentral and acrostichal setae ending in anterior two thirds of scutum. Posterior sides of scutum silvery shining. Pleural sides of thorax without setae, silvery grey coloured. All thoracic setae black. Uniserial row of acrostichal setae, two rows of about 10 dorsocentral setae. Humeral callus with top brownish, with 2 humeral setae; 4 small posthumeral setae. One postalar seta. Notopleural group composed of 6 setae: 1^st^ long, 2^nd^– 4^th^ short, 5^th^–6^th^ long. Notopleural area silvery grey dusted. Two long presutural and 4–5 small postsutural setae. Scutellum black, with 2 prominent scutellar setae on each side. Haltere brownish with knob black.

*Wing* hyaline with brown to dark brown veins. Subcostal cell (sc) yellow tinted and with microtrichia. Wing surface not uniformly covered with microtrichia, microtrichia present on anal lobe, posterior and distal part of wing. First longitudinal vein (R_1_) bearing 9–10 spines. Anterior (r-m) and posterior (dm-cu) crossveins present. Costal cell (c) equal to sc in length. Posterior crossvein (dm-cu) twice as long as distal part of the fifth longitudinal vein (CuA_1_). Anal cell (cup) elongated, its length about three times portion of anal vein (A_1_+CuA_2_) beyond it.

*Legs* slender, brown, slightly silvery shiny. All coxae silvery dusted with black setae, yellow distally. Fore femur with longer fine ventral setae distally. Fore femur with 1 oxhorn seta. Apices of femora and basal parts of tibiae (=“knees”) yellow. Fore tibia with 1 anteroventral spur. Fore tarsomeres I−II yellow. Mid tibia bearing short dorsal seta above middle (anterodorsal seta absent) and two long ventral apical spurs. Hind femur of the same width as hind tibia. Hind tarsomere I with ventral seta above middle.

*Abdomen* black with silver-grey coloured markings. Setae on abdomen fine and black. Tergites 1 and 2 (T1+2) more setulose than the others, T3+T4 sparsely setulose. T1 black, its anterior half shiny silvery grey in lateral view. T2 black with silvery grey marking on posteroventral area. In lateral view this marking occupies posterior third of T2. T3 black, with similar (but smaller) marking, mainly on ventral part. T4 black, with silvery grey marking on posteroventral area occupying posterior two thirds in lateral view. T5 black. T6 black with posterior border grey. T7 small, entirely grey. Sternite 8 also grey, without setae.

*Genitalia* (Figure 10) with epandrium grey, cercus brownish, surstylus and hypandrium shiny amber-brown. Paramere (postgonite) slightly curved dorsally with base narrower, its broader apical part slightly tapered towards the rounded apex. Aedeagus (phallus) broad in lateral view, its dorsal apex with sharp tooth anteriorly, bluntly rounded posteriorly. Hypandrium with small inner hypandrial lobe (ihl) sub-basal to gonopod. Gonopod (hypandrial lobe) trifid, with shorter basal gonopodal lobe (bgl) and longer terminal part deeply bifid forming two slender apical lobes. Surstylus with basal part narrower than its apical rounded part, the latter with a digitiform dorsal process. Terminal lobe of epandrium gradually tapered and slightly curved, covered by setulae and with 2 longer setae. Ventral part of epandrium with 5 prominent setae two smaller setae and two additional smaller setae positioned more ventrally. Cercus covered by microtrichia and with short curly setulae on apex.

***Female.*** Not studied, for description see Chandler (1976, 2001).

###### Distribution.

Palaearctic species. Hitherto recorded only from Bulgaria (Chandler 1976) and the Far East of Russia (Shatalkin 1985). **New record for Romania.**

###### Biology.

Unknown. The larvae of other European *Callomyia* species develop on mycelia under bark of various trees (see above under *Callomyia amoena*). The adult male examined was swept from vegetation close to a cave along a small brook (Figure 17). The known flight period in Europe is in VI.

###### Comments.

This is the second specimen and first male of the species from Europe. Other known material (♂♂ and ♀♀) was collected in the Far East of Russia, Amur region (Shatalkin 1985). It is similar to *Callomyia dives* in the morphology of males, but the silver coloration on the thorax and abdomen is less developed. Also, the basal lobe of the gonopod (bgl) is shorter (Figure 10). This species seems to have an inland distribution, whereas *Callomyia dives* is found mostly on islands or close to coastal zones of Europe.

Chandler (2001) figured genitalia of an Amur specimen collected by Shatalkin. However, the genitalia of our specimen do not entirely fit into the description and figure of Chandler (2001). The main differences appear to be as follows (see Figure 10): presence of inner hypandrial lobe (ihl) positioned sub-basally to gonopod (this character is present in more *Callomyia* species, but usually omitted in the descriptions and figures in the literature); paramere (postgonite) is wider in lateral view, curved dorsally, with narrow basal part; morphology and position of basal gonopodal lobe (bgl) is very similar to that of Chandler (2001), but the bifurcation of terminal part of gonopod is more profound; the apical part of surstylus is less rounded and its basal part is only slightly narrower than apical part; cerci have shorter curly setulae.

**Figure 9.**
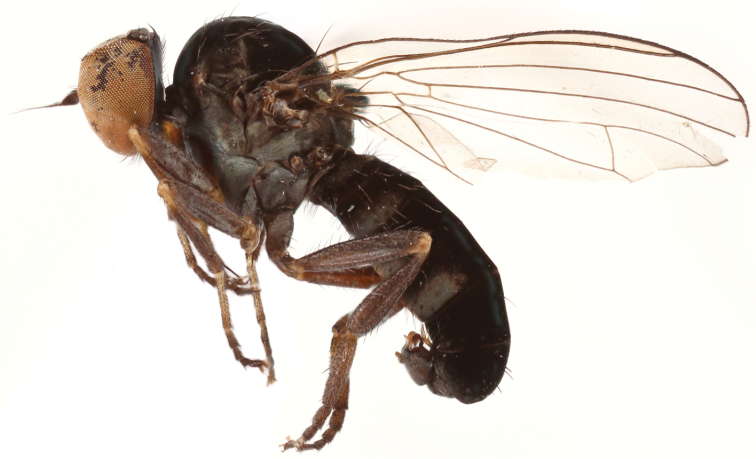
*Callomyia saibhira* Chandler, 1976, male from Romania: body in lateral view. Photo by M. Tkoč.

**Figure 10.**
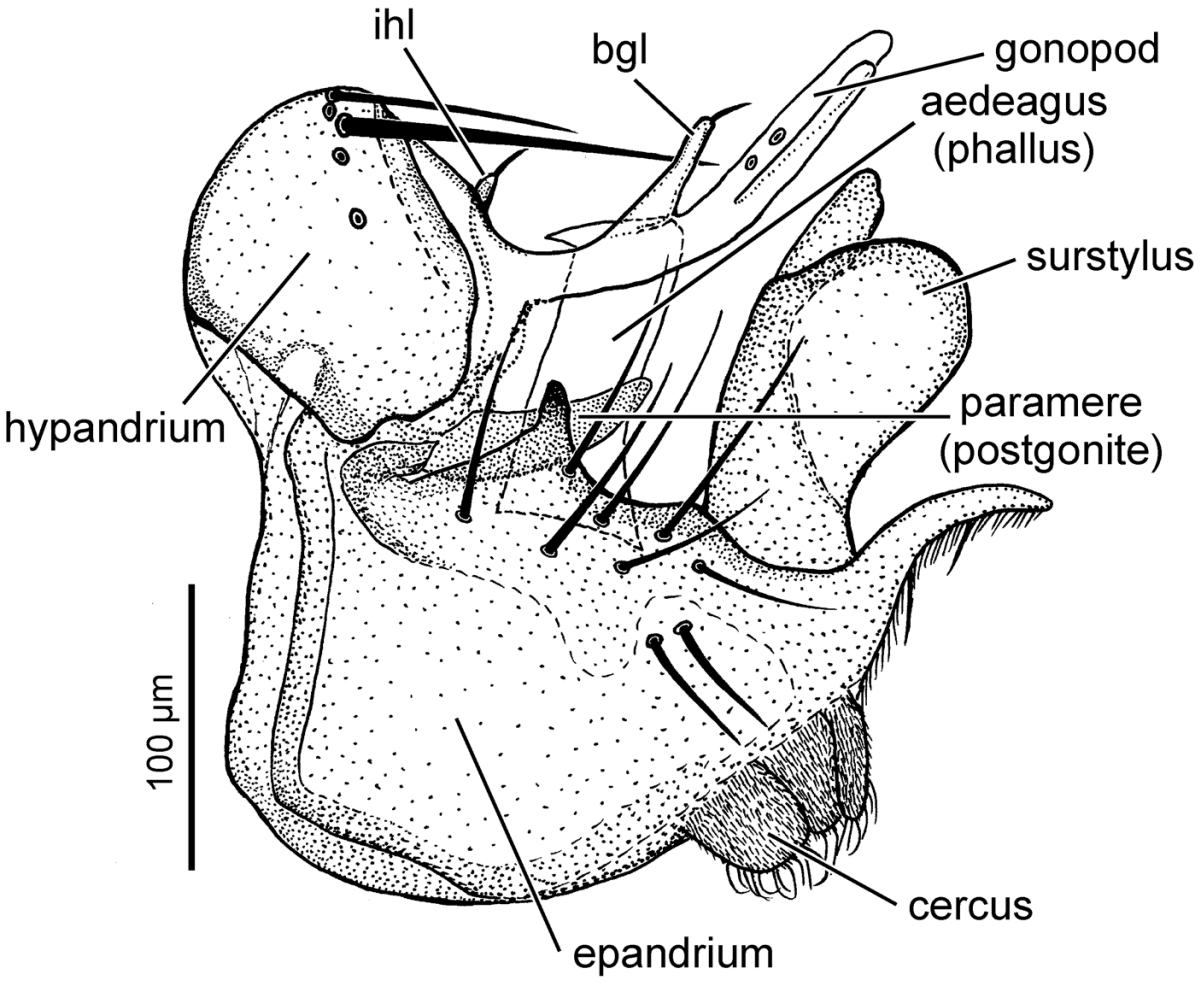
*Callomyia saibhira* Chandler, 1976, male genitalia of specimen from Romania: right lateral view. (bgl – basal gonopodal lobe, ihl – inner hypandrial lobe).

##### Callomyia speciosa

Taxon classificationAnimaliaDipteraPlatypezidae

Meigen, 1824

Figure 11

###### Published records.

1 ♂, Retezatului Mts, near Hobita, Calana [Hobița, Hunedoara], 29. vi. 1969, in mature pine forest, B.H. Cogan and R.I. Vane-Wright leg. (BMNH) (Chandler 2001, in litt.).

###### Material examined.

1 ♀, 31. v. 2011, Banat, Sfânta Elena, 1 km E, Alibeg brook valley (Figure 18), 230 m a.s.l., 44°40'37"N, 21°43'32"E, sweeping undergrowth of deciduous forest, JR leg.; 1 ♂, 1 vi. 2008, Banat, Sfânta Elena, 4 km NE, Kulhavá skála, Vranovec cave (Figure 17), 300 m a.s.l., 44°42'12"N, 21°43'52"E, sweeping vegetation along brook, JR leg.; 1 ♀, 3. vi. 2008, Banat, Sfânta Elena, 1 km E, Alibeg brook valley (Figure 18), 230 m a.s.l., 44°40'37"N, 21°43'32"E, sweeping vegetation along brook, JR leg.

###### Distribution.

Palaearctic species. Recorded in Andorra, Austria, Belgium, the Czech Republic, Denmark, Finland, France, Germany, Great Britain, Greece, Hungary, Italy, Israel, Lithuania, the Netherlands, Norway, Poland, Portugal, Romania, Slovakia, Slovenia, Spain, Sweden, Switzerland, Turkey, Russia (ER) and Caucasus.

###### Biology.

Less common than *Callomyia amoena* but widely distributed throughout Europe. The larvae also live on mycelia under bark of various trees (reared from mycelium on surface of a fallen hazel trunk by Krivosheina (2008)). Adults fly in V–IX.

**Figure 11.**
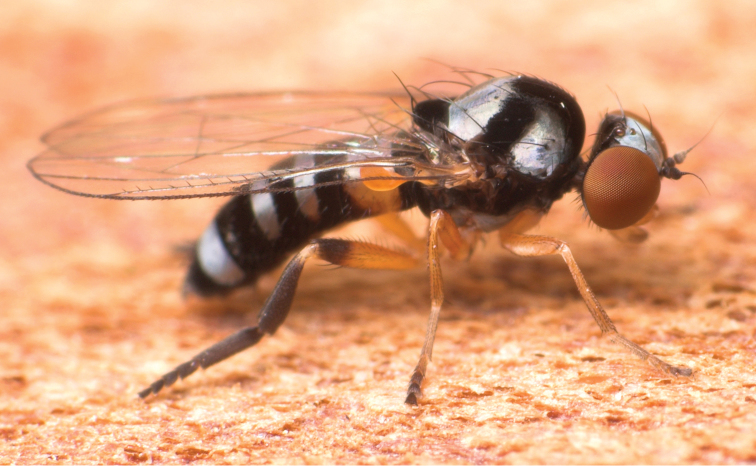
*Callomyia speciosa* Meigen, 1824, female habitus. Photo by D. Gavryushin.

#### Subfamily Platypezinae

##### Seri obscuripennis

Taxon classificationAnimaliaDipteraPlatypezidae

(Oldenberg, 1916)

Figure 12

###### Published records.

1 ♂, Herkulesbad [Băile Herculane, Caraș-Severin, Banat], 6.vi.1904, Kertész lgt. (Oldenberg 1916, Czerny 1930); Mehádia [Mehadia, Caraș-Severin, Banat] (Szilády 1941).

###### Distribution.

Palaearctic species. Recorded in Austria, the Czech Republic, Finland, Germany, Great Britain, Hungary, the Netherlands, Norway, Poland, Romania, Slovakia, Sweden, Switzerland and Russia (ER, FE).

###### Biology.

Rather rare in Europe but more common in the Far East of Russia (Shatalkin 1985, Chandler 2001). Its larvae develop in several species of *Polyporus* (Ševčík 2010). Adults occur in VI–VII and IX.

**Figure 12.**
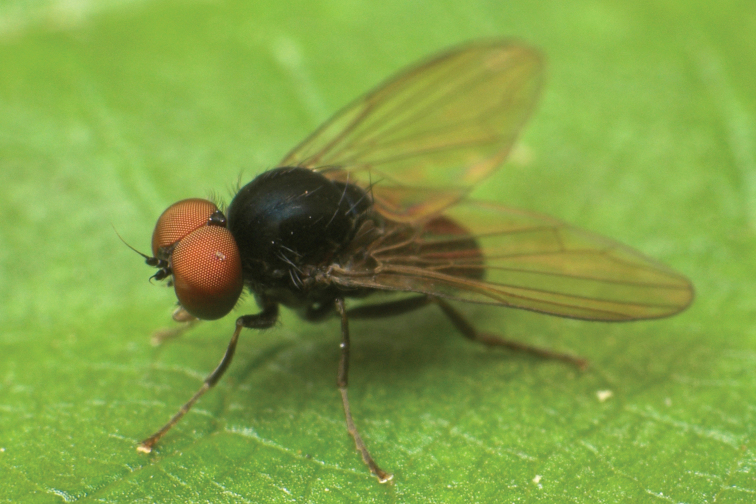
*Seri obscuripennis* (Oldenberg, 1916), male habitus. Photo by D. Gavryushin.

##### Bolopus furcatus

Taxon classificationAnimaliaDipteraPlatypezidae

(Fallén, 1826)

###### Published records.

Mehádia [Mehadia, Caraș-Severin] (Szilády 1941).

###### Distribution.

Palaearctic species. Recorded in Austria, Belgium, the Czech Republic, Denmark, Finland, Germany, Great Britain, Hungary, Ireland, Poland, Romania, Slovakia, Sweden, Switzerland, the Netherlands, Russia (ER).

###### Biology.

Immature stages develop in *Polyporus squamosus* (Chandler 2001, Ševčík 2010). Adult flight period is from IV to IX.

##### Polyporivora ornata

Taxon classificationAnimaliaDipteraPlatypezidae

(Meigen, 1838)

Figure 13

###### Published records.

Szászka [Szászka, Caraș-Severin, Banat] (Szilády 1941).

###### Material examined.

1 ♂, 19. v. 2011, Alba, Alba Iulia, 1 km E, 380 m a.s.l., 46°04'18"N, 23°32'02"E, sweeping on *Fagus sylvatica*, MT leg.

###### Distribution.

Palaearctic species. Recorded in Belgium, the Czech Republic, Denmark, Finland, France, Germany, Great Britain, Hungary, Ireland, Italy, Lithuania, the Netherlands, Norway, Poland, Portugal, Romania, Slovakia, Spain, Sweden, Switzerland and Russia (ER).

###### Biology.

Immature stages develop in *Trametes versicolor* (Ševčík 2010). Adult flight period ranges from V to X, the species is bivoltine.

**Figure 13.**
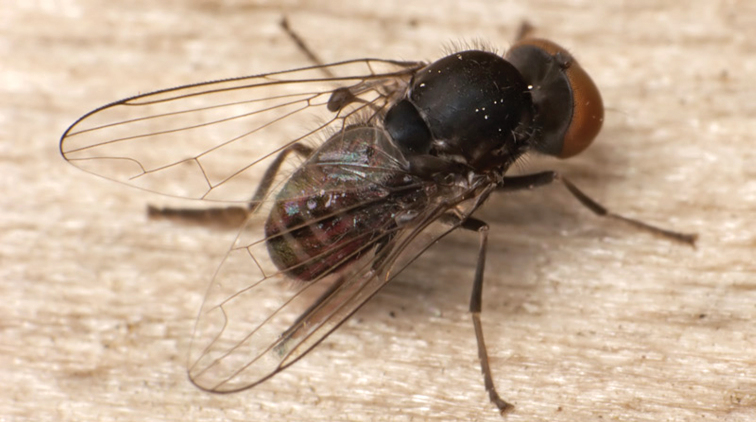
*Polyporivora ornata* (Meigen, 1838), male habitus. Photo by D. Gavryushin.

##### Paraplatypeza atra

Taxon classificationAnimaliaDipteraPlatypezidae

(Meigen, 1804)

###### Published records.

Mehádia [Mehadia, Caraș-Severin, Banat] (Thalhammer 1899); Mehádia [Mehadia, Caraș-Severin, Banat], Radna-Borberek [Rodna, Bistrița-Năsăud, Transilvania] (Szilády 1941).

###### Material examined.

1 ♂, 1. vi. 2008, Banat, Sfânta Elena, 4 km NE, Kulhavá skála, Vranovec cave (Figure 17), 300 m a.s.l., 44°42'12"N, 21°43'52"E, sweeping vegetation along brook, JR leg.; 1 ♀, 18. v. 2011, Alba, Alba Iulia, 1 km E, 380 m a.s.l., 46°04'18"N (46.07168), 23°32'02"E (23.53429), sweeping on *Fagus sylvatica*, K. Blahová & MT leg.

###### Distribution.

Palaearctic species. Recorded in Austria, Belgium, the Czech Republic, Denmark, Finland, France, Germany, Great Britain, Hungary, Ireland, Italy, Lithuania, the Netherlands, Norway, Poland, Portugal, Romania, Slovakia, Sweden, Switzerland and Russia (ER).

###### Biology.

Common species, larvae develop in various species of *Pluteus*, mainly in *Pluteus cervinus* (Ševčík 2010). Adult flight period ranges from IV to XI.

##### Paraplatypeza bicincta

Taxon classificationAnimaliaDipteraPlatypezidae

(Szilády, 1941)

Figure 14

###### Published records.

Szászka [Szászka, Caraș-Severin, Banat] (Szilády 1941).

###### Distribution.

Palaearctic species reaching to Oriental region. Distributed in the Czech Republic, Great Britain, Finland, Norway, Slovakia, Switzerland, Sweden, Russia and Myanmar [=Burma].

###### Biology.

Rare species associated with *Pluteus* sp. The adults were reared from *Pluteus cervinus* several times by the first author (not published). Adult flight period ranges from VIII to X.

**Figure 14.**
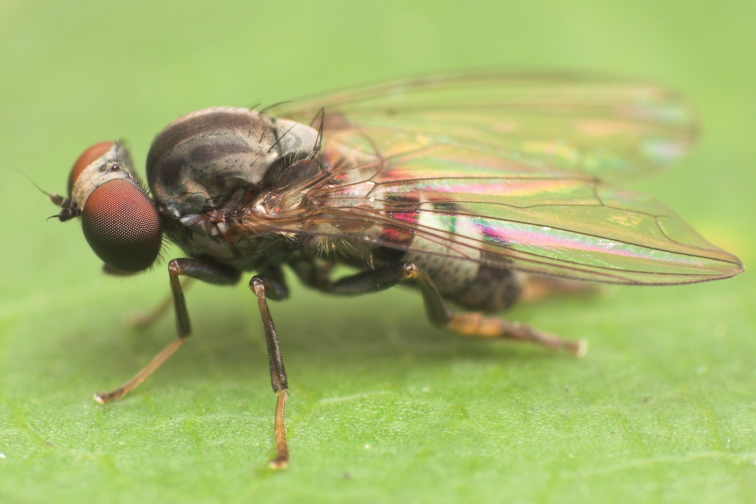
*Paraplatypeza bicincta* (Szilády, 1941), female habitus. Photo by D. Gavryushin.

##### Lindneromyia dorsalis

Taxon classificationAnimaliaDipteraPlatypezidae

(Meigen, 1804)

Figure 15

###### Published records.

1♂, „Bucarest” [București], A.L. Montandon leg., ex E. Brunetti coll. (BMNH) (Chandler 2001, in litt.).

###### Material examined.

1 ♂, 25. v. 2013, Mer occ., Muntii Locvei Mts., Sfanta Elena env., cca 44°40'N, 21°43'E, B. Mocek leg.; 1 ♀, 30. v. 2008, Banat, Latunas, 3 km W nr. Comoraste, 110 m a.s.l., 45°13'16"N, 21°28'10"E, sweeping over boggy meadow, JR leg.; 1 ♂, 1. vi. 2008, Banat, Sfânta Elena, 4 km NE, Kulhavá skála, Vranovec cave (Figure 17), 300 m a.s.l., 44°42'12"N, 21°43'52"E, sweeping vegetation along brook, JR leg.; 1 ♀, 19. v. 2011, Alba, Alba Iulia, 1 km E, 380 m a.s.l., 46°04'18"N (46.07168), 23°32'02"E (23.53429), sweeping on *Fagus sylvatica*, MT leg.; 1 ♀, 19. v. 2011, Alba, Alba Iulia, 1 km E, 380 m a.s.l., 46°04'18"N (46.07168), 23°32'02"E (23.53429), sweeping on *Fagus sylvatica*, MT leg.; 2 ♀, 18. v. 2011, Alba, Alba Iulia, 1 km E, 380 m a.s.l., 46°04'18"N (46.07168), 23°32'02"E (23.53429), sweeping on *Fagus sylvatica*, K. Blahová & MT leg.

###### Distribution.

Palaearctic species. Recorded in Andorra, Austria, Belgium, Cyprus, the Czech Republic, Denmark, France, Germany, Great Britain, Greece, Hungary, Italy, Israel, Morocco, the Netherlands, Poland, Portugal, Romania, Slovakia, Spain, Switzerland, Turkey and Russia (ER).

###### Biology.

Larvae mostly develop in fruiting bodies of various *Agaricus* sp. There are several unconfirmed records from other soft fungi (Chandler 2001). The adult females may be observed during oviposition directly on fruiting bodies. Adults occur in V–XI.

**Figure 15.**
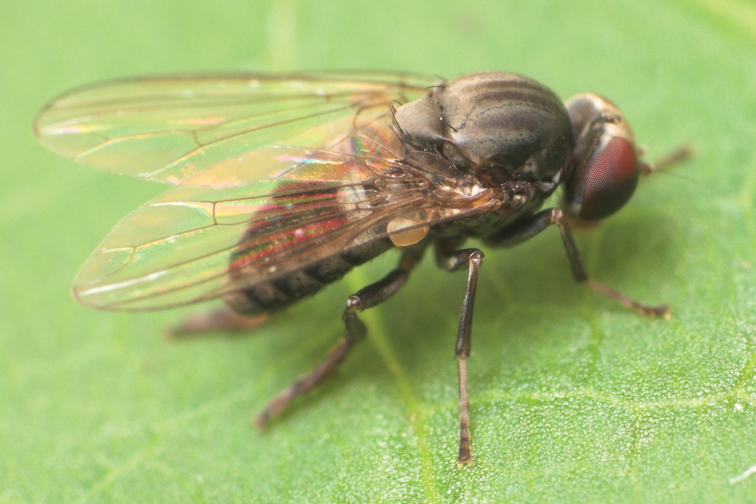
*Lindneromyia dorsalis* (Meigen, 1804), female habitus. Photo by D. Gavryushin.

##### * Lindneromyia hungarica

Taxon classificationAnimaliaDipteraPlatypezidae

Chandler, 2001

Figure 16

###### Material examined.

1 ♀, 1. vi. 2008, Banat, Sfânta Elena, 2.5 km NE, 420 m a.s.l., 44°41'44"N, 21°43'10"E, sweeping over meadow, JR leg.

###### Distribution.

Palaearctic species. Recorded in Austria, the Czech Republic, France, Germany, Great Britain, Hungary, Portugal, Slovakia, Spain and Switzerland. **New record for Romania.**

###### Biology.

The species was only recently separated from *Lindneromyia dorsalis* and their larvae can develop together with those of *Lindneromyia dorsalis* in the same fruiting body of an *Agaricus* sp. (Chandler 2001, Tkoč and Vaňhara 2008). Occasionally, the species can be caught on sporocarps of other fungi, e.g. Roháček and Ševčík (2013) collected one female on *Meripilus giganteus* in a park of Opava city (Czech Republic). Adults can be found in V–X.

**Figure 16.**
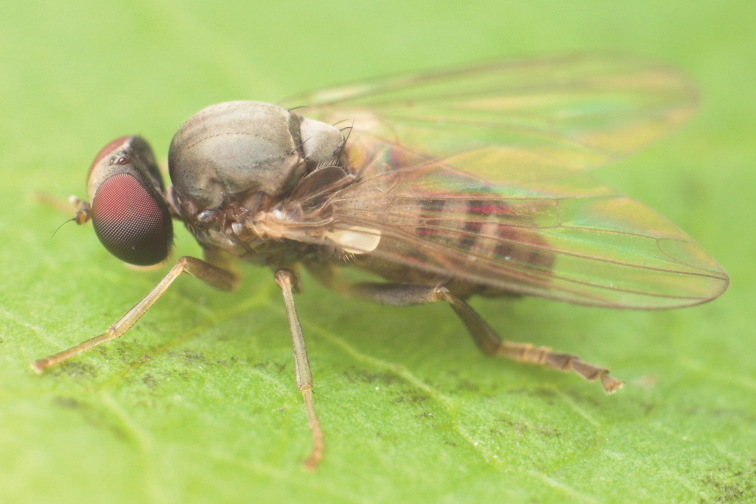
*Lindneromyia hungarica* Chandler, 2001, female habitus. Photo by D. Gavryushin.

### Checklist of the Romanian Opetiidae and Platypezidae

Family Opetiidae

*Opetia nigra* Meigen, 1830

Family Platypezidae

Subfamily Callomyiinae

*Agathomyia antennata* (Zetterstedt, 1819)

*Agathomyia collini* Verrall, 1901

*Agathomyia falleni* (Zetterstedt, 1838)

*Agathomyia setipes* Oldenberg, 1916

*Agathomyia vernalis* Shatalkin, 1981

*Agathomyia viduella* (Zetterstedt, 1838)

*Callomyia amoena* Meigen, 1824

*Callomyia elegans* Meigen, 1804

*Callomyia saibhira* Chandler, 1976

*Callomyia speciosa* Meigen, 1824

Subfamily Platypezinae

*Seri obscuripennis* (Oldenberg, 1916)

*Bolopus furcatus* (Fallén, 1826)

*Polyporivora ornata* (Meigen, 1838)

*Paraplatypeza atra* (Meigen, 1804)

*Paraplatypeza bicincta* (Szilády, 1941)

*Lindneromyia dorsalis* (Meigen, 1804)

*Lindneromyia hungarica* Chandler, 2001

## Discussion

Altogether 18 species of the families Opetiidae and Platypezidae are reported from Romania, representing 40.9 % of all flat-footed flies known from Europe. This number is far from the total number of species in this country and more research on flat-footed flies is needed to understand their distribution in Romania and Europe as a whole. Comparing the species number of the family Opetiidae and Platypezidae from Romania with the countries related to the Carphathian-Pannonian region it is an average number of species: Austria has 17 species (Chandler 2004), the Czech Republic 34 (Vaňhara 2009), Hungary 27 (Papp 2001, Weele 2001, Chandler 2004), Poland 25 (Chandler 2004), Slovakia 34 (Vaňhara 2009); the flat-footed fly fauna of Croatia, Serbia, Slovenia and Ukraine has not been systematically studied, thus the numbers are very low and not useful for comparison.

The discovery of the first male of *Callomyia saibhira* in Romania is the most important result of this study. The genitalia figured herein do not entirely agree with the description and figure of Chandler (2001). The differences in male genitalia between the Romanian and Amur specimen highlighted above may be the result of simple variation, or could be caused by comparing our specimen (Figure 10) with the somewhat simplified illustration of Chandler (2001) or may point to a more complicated taxonomic problem. To resolve this question, additional fresh material from both Europe and the Far East of Russia is needed in order to study variability in the morphology of the male genitalia and to test for differences using molecular methods. It also needs to be determined, if the males assigned to this species are correctly associated with the females, which appears to be a problem in the Nearctic species of *Callomyia* (Chandler 2001; Tkoč 2012).

**Figures 17–18.**
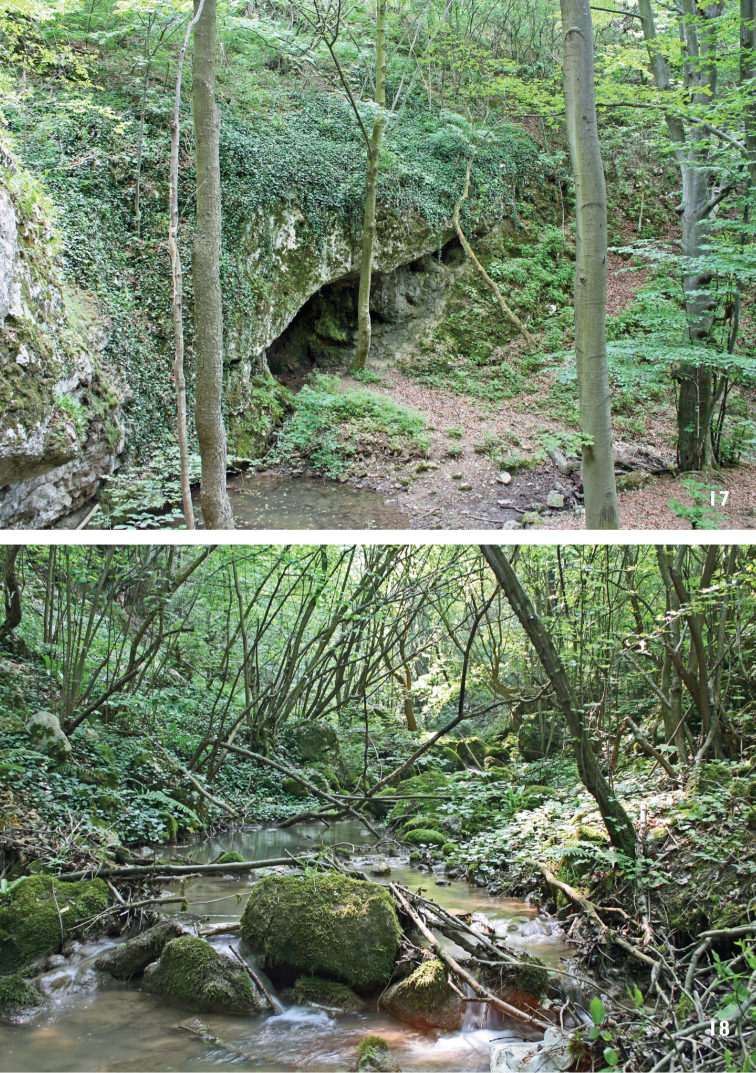
Habitats of Opetiidae and Platypezidae in Romania: **17** Kulhavá skála, Vranovec cave **18** Alibeg brook valley. Photos by J. Roháček.

## Supplementary Material

XML Treatment for Opetia nigra

XML Treatment for Agathomyia antennata

XML Treatment for Agathomyia collini

XML Treatment for Agathomyia falleni

XML Treatment for Agathomyia setipes

XML Treatment for Agathomyia vernalis

XML Treatment for Agathomyia viduella

XML Treatment for Callomyia amoena

XML Treatment for Callomyia elegans

XML Treatment for Callomyia saibhira

XML Treatment for Callomyia speciosa

XML Treatment for Seri obscuripennis

XML Treatment for Bolopus furcatus

XML Treatment for Polyporivora ornata

XML Treatment for Paraplatypeza atra

XML Treatment for Paraplatypeza bicincta

XML Treatment for Lindneromyia dorsalis

XML Treatment for Lindneromyia hungarica
